# 2139: PBX1 mRNA expression is a prognostic biomarker of and clinical indicator by RT-qPCR and RNAScope^®^ in situ hybridization in neuroblastoma

**DOI:** 10.1017/cts.2017.205

**Published:** 2018-05-10

**Authors:** Nilay Shah, Julia Selich-Anderson

**Affiliations:** The Ohio State University, Columbus, OH, USA

## Abstract

OBJECTIVES/SPECIFIC AIMS: (1) Correlate PBX1 mRNA expression as measured by RNAScope in situ hybridization, at an RNA number/cell measurement, Versus by RT-qPCR by the ddCt method. (2) Validate PBX1 mRNA expression in a second independent cohort of neuroblastoma tumor samples, and correlate with patient outcomes. We expect that PBX1 expression will correlate whether detected by RNAScope or by RT-qPCR. This work has the promise of validating a novel biomarker of disease severity, and for clinical translation as the RNAscope technology has been CLIA-certified for clinical use for other genes. METHODS/STUDY POPULATION: Primary neuroblastoma tumor samples were acquired through the Children’s Oncology Group Tumor Bank, The Cooperative Human Tissue Network Tumor bank, and the Westmeade Tumor Bank (Westmeade, Australia), with patient outcomes annotated but sequestered until experiments are completed. RT-qPCR is performed using 1 μg total RNA isolated from each sample by Nucleospin RNA kit (Clontech), reverse transcribed by SuperScript VILO (ThermoFisher Scientific) and amplified using KiCqSTART SYBR Green qPCR mix (Sigma Aldrich). RNAScope was performed on sections of fresh frozen tumor, in triplicate, per manufacturer protocol (ACDBio) using company-designed probes. Statistical analyses performed using GraphPad Prism5. RESULTS/ANTICIPATED RESULTS: PBX1 mRNA expression as measured RNAScope correlated well with matched RT-qPCR values, with most PBX1 transcripts identified within the malignant cells and not in tumor stroma. Correlation with patient outcomes is ongoing (expected to be available at the time of presentation), but as the RNAScope values correlate with *R*>0.9 with RT-qPCR values, we expect good correlation with outcomes in our primary data set and matching validation set. DISCUSSION/SIGNIFICANCE OF IMPACT: PBX1 mRNA expression is an accurate prognostic biomarker of outcome in low and intermediate-risk neuroblastoma, and testing on an additional validation set is planned based on thresholds established by RNAScope. RNAScope is a method readily translatable to clinical use and its inclusion in future clinical trials will be further studied. It provides an additional benefit that concomitant immunohistochemistry can also be performed. Analysis of high-risk neuroblastomas for responsiveness to retinoic acid based on PBX1 expression is planned.

